# Explosive hamstrings strength asymmetry persists despite maximal hamstring strength recovery following anterior cruciate ligament reconstruction using hamstring tendon autografts

**DOI:** 10.1007/s00167-022-07096-y

**Published:** 2022-08-23

**Authors:** Argell T. San Jose, Nirav Maniar, Ryan G. Timmins, Kate Beerworth, Chris Hampel, Natalie Tyson, Morgan D. Williams, David A. Opar

**Affiliations:** 1 School of Behavioural and Health Sciences, Australian Catholic University, Melbourne, VIC Australia; 2 Sports Performance, Recovery, Injury and New Technologies (SPRINT) Research Centre, Australian Catholic University, Fitzroy, VIC Australia; 3 Wakefield Sports and Exercise Medicine Clinic, Adelaide, SA Australia; 4 Cricket Australia, Albion, QLD Australia; 5 School of Health, Sport and Professional Practice, University of South Wales, Pontypridd, Wales UK

**Keywords:** Anterior cruciate ligament reconstruction, Strength, Rate of torque development, Hamstring, Quadriceps

## Abstract

**Purpose:**

To investigate the differences in maximal (isometric and concentric peak torque) and explosive (rate of torque development (RTD)) hamstring and quadriceps strength symmetry between males and females during early- and late-phase rehabilitation after anterior cruciate ligament reconstruction (ACLR) using hamstring tendon (HT) autografts and to determine the interaction of time and sex on maximal and explosive strength symmetry.

**Methods:**

A total of 38 female and 51 male participants were assessed during early (3–6 months post-operative) and late (7–12 months post-operative) phases of rehabilitation following ACLR. Maximal (concentric and isometric peak torque) and explosive (isometric RTD) hamstring and quadriceps strength were assessed and presented as limb symmetry index (LSI).

**Results:**

Maximal concentric hamstrings asymmetry (Early: 86 ± 14; Late 92 ± 13; *p* = 0.005) as well as maximal concentric (Early, 73 ± 15; Late 91 ± 12; *p* < 0.001) and explosive (Early: 82 ± 30; Late: 92 ± 25; *p* = 0.03) quadriceps asymmetry decreased from early to late rehabilitation. However, there were no significant changes in maximal isometric quadriceps strength and explosive isometric hamstring strength in the same time period. Females had a larger asymmetry in maximal concentric (Females: 75 ± 17; Males: 81 ± 15; *p* = 0.001) and explosive (Females: 81 ± 32; Males: 89 ± 25; *p* = 0.01) quadriceps strength than males throughout rehabilitation. There were no sex differences in maximal and explosive hamstring strength. There were no sex by time interactions for any variables.

**Conclusion:**

Explosive hamstring strength asymmetry did not improve despite recovery of maximal hamstring strength during rehabilitation following ACLR with HT autografts. While sex did not influence strength recovery, females had larger maximal and explosive quadriceps strength asymmetry compared to males throughout rehabilitation following ACLR.

**Level of evidence:**

Level III

**Supplementary Information:**

The online version contains supplementary material available at 10.1007/s00167-022-07096-y.

## Introduction

Anterior cruciate ligament (ACL) ruptures are traumatic injuries that commonly occur during jumping, cutting, and pivoting sports [1]. There is an increase in ACL injury rates in the last 15 years [2], with females reported to be at higher relative risks when compared to males [1]. Surgical management with ACL reconstruction (ACLR), a protracted rehabilitation period (6–12 + months), and financial costs between $100 million [2] to $2 billion annually [3] makes ACL injuries burdensome. However, despite ACLR and rehabilitation, poor outcomes related to return to sports [4], recurrent ACL injury [5], and knee osteoarthritis following the injury [6] are commonly reported. These poor outcomes have been reported to be worse in females than males [7, 8], suggesting a potential sex influence in outcomes following ACLR.

Hamstrings and quadriceps strength asymmetry are common after ACLR and have been previously reported to be graft-related (e.g., hamstring strength asymmetries more common with hamstring tendon graft use) [9]. These asymmetries are critical given the role of the hamstrings and quadriceps to knee joint stability [10]. Hamstring and quadriceps strength is commonly measured during maximal isometric or isokinetic contractions to assess strength recovery [11]. This is typically reported as between-limb strength or limb symmetry index (LSI) [12]. Greater levels of between leg asymmetry in hamstring and/or quadriceps strength is associated with poorer patient reported outcomes [13] and alterations in function and performance [13, 14]. Greater between leg quadriceps asymmetry has also been linked to an increased risk of re-injury [12]. Consequently, one of the main foci of rehabilitation and subsequent return to sports following ACLR is the recovery of hamstrings and quadriceps strength symmetry.

Recent evidences have shown that rate of torque development (RTD) or explosive strength might be an important criterion to assess strength recovery after ACLR [15]. Explosive strength is associated with knee function [16] and lower limb kinetics [17] following ACLR. Like maximal strength, explosive strength asymmetries are also found during the early (< 6 months) [15, 17] and late phase (> 7–12 months) [15] of rehabilitation following ACLR. However, there is some evidence that explosive quadriceps strength does not recover at the same rate as maximal quadriceps strength [18]. It is still inconclusive whether the same pattern of recovery exists in maximal and explosive hamstring strength. Given the common use of hamstring grafts, investigating explosive hamstring strength recovery in patients who had ACLR using hamstring tendon grafts is important to inform exercise selection during rehabilitation.

There is some evidence that maximal hamstring and quadriceps strength asymmetry following ACLR is more pronounced in females [16]. Kuenze et al. [16] found that females have larger explosive quadriceps strength asymmetries compared to males. However, it is still not known whether females also have significant asymmetries in explosive hamstrings strength following ACLR. Additionally, whether recovery of maximal and explosive hamstrings and quadriceps strength differs between males and females is still unknown. Given the poorer outcomes in females and the potential implications of both maximal and explosive strength to these outcomes, understanding how males and females may recover differently during rehabilitation is an important first step in advancing the knowledge base in this area.

Therefore, the aims of this observational cohort study are (1) to investigate the effect of time on maximal and explosive hamstring and quadriceps strength asymmetry during the early and late phase of rehabilitation following ACLR using HT autografts, and (2) to explore the effect of sex on these asymmetries.

## Materials and methods

This study was approved by the Australian Catholic University Human Research Ethics Committee (approval number: 2017–17HC). The study utilized an observational cohort study design with data collected from a community-based clinic. Participants were recruited from a sports clinic between 2017 and 2018. These patients had suffered a primary ACL rupture and underwent a subsequent ACLR performed using either a semitendinosus tendon autograft (*n* = 47) or a semitendinosus with gracilis tendon autograft (*n* = 42) by 10 surgeons from the same clinic and one surgeon from another practice.

Recruited participants were assessed between 3–6 months (early rehabilitation period) and 7–12 months (late rehabilitation period) following their ACLR. These time points were utilized as six and 12 months are the most commonly used cut-off for return to sports participation (six months) [19] and full clearance to return to sports (12 months) [20]. There was a total of 89 patients assessed during the early rehabilitation phaseand 42 were re-assessed during the late rehabilitation phase. Rehabilitation was designed and prescribed by physiotherapists both from, and external, to the clinic in consultation with the patient’s surgeon. Of the 89 patients, 48 had their rehabilitation supervised at the clinic while the remaining 41 patients undertook supervised rehabilitation programs from physiotherapists external to the clinic, but guidance was provided to the external physiotherapists on the rehabilitation approached used by the clinic. The rehabilitation protocol from the clinic included primary outcome measures related to: management of swelling/effusion; early restoration of knee extension and flexion; early ambulation; and recovery of hamstrings and quadriceps strength through gradual and progressive overload using open- and close-chain kinetic exercises [21]. Participants were progressed to running, plyometric training, and sports specific activities based on their progression from the aforementioned outcome measures [21].

Inclusion criteria were (1) age 15–40 years; (2) primary ACL injury; (3) unilateral ACLR using hamstring tendon autograft (semitendinosus only or semitendinosus with gracilis) taken from the injured leg; (4) ACLR within the previous three to six months prior to first testing session. Patients who had a previous ACLR or any other major knee joint/ligament injury were excluded. All participants provided written informed consent (or for minors, consent was provided by their guardian and the minor provided assent) to be included in the study.

### Procedures

Data collection was performed during the participant’s routine physiotherapy consultation in the clinic. All subjective and clinical assessments of the knee joint were collected during these visits which occurred between 3–6 months and 7–12 months after ACLR. Upon arrival at the clinic, participants completed the International Knee Documentation Committee (IKDC) which have been previously reported to be a valid and reliable measure of the participants’ perception of their knee function and activities of daily living [22]. The participants then performed a strength testing battery for the hamstrings and quadriceps using an isokinetic dynamometer followed by a single leg hop for distance test [23].

Maximal voluntary contractions (MVC) of the hamstrings and quadriceps during isometric and concentric contractions were collected during the trials. Prior to strength testing, participants were asked to perform a 5-min warm-up on a cycle ergometer using low resistance. Participants were then seated on a Humac Isokinetic Dynamometer (CSMi, Stoughton, MA, USA) with hips maintained at 85° of flexion throughout the test and the lateral epicondyle of the femur aligned with the fulcrum of the dynamometer. Correction for limb weight was taken before the test [24]. All dynamometer strength tests were conducted on each leg, commencing with the uninjured leg. Verbal encouragement and visual feedback was provided by the tester on all tests to motivate the participant to perform maximally throughout all measurements [24].

Isometric hamstrings and quadriceps strength testing commenced with the participant’s knee 45° from full knee extension. The participant was instructed to pull the leg down (hamstring assessment) as hard and as fast as possible for three seconds, rest for two seconds, and then push the lower leg up (quadriceps assessment) as hard and fast as possible for three seconds. This was performed for three repetitions per muscle group [24]. The participant then rested for two minutes while seated on the dynamometer. While resting, range of motion for concentric strength testing was set at 0–90° of knee flexion (0° = full extension). For the concentric strength test, participants performed five repetitions of knee extension and flexion at 180°/sec, followed by 60°/sec. Participants performed one set of five consecutive MVCs at each movement velocity with a minimum of 30 s of rest between sets. Isokinetic/isometric strength testing is the gold standard for strength assessment following ACLR [11] and has been previously shown to be reliable (ICC = 0.81–0.97) [25].

After isokinetic strength testing, participants were then asked to perform a single leg hop for distance [23]. The test was performed by the participants barefooted, starting from a stationary position in a single leg stance with their hands placed on their lower back. The participant then performed a single hop for maximum distance, landing on the same leg [23]. The participant was required to hold their final landing position without their contralateral limb and/or their upper limbs touching down on the floor for the test to be considered successful. This was performed three times on each leg with the best score from three trials recorded in centimeters by measuring the distance covered from the line of the great toe before and after the hop [23].

### Data reduction

A custom-written software program was used to collect data (LabVIEW 2017 SP1; National Instruments, Austin, TX) from the isokinetic dynamometer with torque and time data captured at 1000 Hz. Once data were collected, another custom-written software package (LabVIEW 2017 SP1; National Instruments, Austin, TX) was used to individually process each set of data (isometric and concentric hamstring and quadricep repetitions) from each limb of each participant.

Peak isometric and concentric hamstring and quadriceps strength as well as isometric RTD were determined for each limb at each testing velocity as the highest torque recorded during all repetitions. Peak RTD was defined as the greatest increase in force (increase in resting force ≥ 4 N from onset of contraction) within a rolling 200 ms window, which has previously been shown to be more reliable than alternative methodologies [26, 27]. We decided to collect RTD from isometric contractions as this was deemed more reliable compared to RTD from isokinetic efforts [26]. The isometric repetition with the greatest RTD (Nm/s) for the hamstrings and quadriceps was used for further analysis [28].

Limb symmetry index was calculated as the percentage of the injured limb relative to the uninjured limb for all strength assessments (MVC and RTD) and single leg hop distance, per the equation below [12].

$$\mathrm{LSI}= \frac{\mathrm{injured\,\, limb\,\, score }}{\mathrm{uninjured \,\,limb\,\, score }} \times 100$$

### Statistical analysis

To compare LSI from hamstring and quadriceps MVCs, RTD and single leg hop distance data between sexes across the early (3–6 months post-operative) and late (7–12 months post-operative) phases of ACL rehabilitation, a linear mixed model fitted with restricted maximum likelihood method was utilized with fixed factors of time (early/late) and sex (male/female) as well as the random factor of participant identification number. Interactions between sex and time were explored when a main effect was identified for both fixed factors. Where main or interaction effects were detected a post-hoc Students t-test was used to identify the differences. Statistical analysis was performed using JMP statistical software (Version 14.2.0 2018; SAS Institute, Cary, NC) with significance set at *p* = 0.05. A convenient sample size was used for this study. Post hoc power analyses determined that when comparing between the early (*n* = 89) and late (*n* = 42) rehabilitation groups with isometric hamstring MVC as the outcome measure (*d* = 0.53), the study had a power of 0.88.

## Results

Participant demographic data, patient reported outcomes, and single leg hop for distance data can be found in Table 1 (early and late phases of rehabilitation) and Table 2 (between males and females).

**Table 1:** Summary of participant demographics, patient reported outcome questionnaires, and single leg hop for distance for all participants during the early and late phases of rehabilitation

	Early rehabilitation (*n* = 89)	Late rehabilitation (*n* = 42)	*p *value
Age (years)	21 (18–25)	20 (18–25)	
Height (cm)	168 ± 6	175 ± 10	
Body mass (kg)	76 (66–86)	72 (66–84)	
Time from ACLR (months)	4 (4–5)	10 (9–11)	
IKDC	64 (59–70)	89 (83–95)	< 0.001*
Single leg hop for distance (LSI)	77 ± 18	94 ± 11	< 0.001*

Data presented as mean ± standard deviation for parametric data or median (interquartile range) for non-parametric data; *ACLR* anterior cruciate ligament reconstruction, *IKDC* International Knee Documentation Committee questionnaire, *LSI* limb symmetry index. ^*^p ≤ 0.05

**Table 2:** Summary of participant demographics, patient reported outcome questionnaires, and single leg hop for distance between males and females during the first assessment

	Females (*n* = 38)	Males (*n* = 51)	*P * value
Age (years)	23 (20–29)	21 (18–25)	n.s
Height (cm)	168 ± 6	182 ± 7	< 0.001*
Body mass (kg)	66 (60–76)	82 (76–91)	< 0.001*
Time from ACLR (months)	5 (4–5)	4 (3–5)	n.s
IKDC	63 (59–69)	64 (60–75)	n.s
Single leg hop for distance (LSI)	85 ± 18	86 ± 16	n.s

Data presented as mean ± standard deviation for parametric data or median (interquartile range) for non-parametric data; *ACLR* anterior cruciate ligament reconstruction, *IKDC* International Knee Documentation Committee questionnaire, *LSI* limb symmetry index. *p ≤ 0.05

### Effect of time

Concentric hamstring MVC and isometric MVC LSI improved as a function of time, but isometric RTD LSI did not (Table 3). In addition, both concentric quadriceps MVC and isometric RTD LSI improved with time, but no change was observed for isometric MVC LSI (Table 3).

**Table 3:** Quadriceps and hamstring strength data in early- and late-stage rehabilitation after anterior cruciate ligament reconstruction collapsed across males and females

Strength measure	LSI (%)		Main effect
	Early rehabilitation	Late rehabilitation	Time
Concentric hamstring MVC (60°/sec)	86 ± 14	92 ± 13	0.005*
Concentric hamstring MVC (180°/sec)	88 ± 12	91 ± 13	0.023*
Isometric hamstring MVC	76 ± 17	84 ± 13	0.003*
Isometric hamstring RTD	86 ± 46	83 ± 22	n.s
Concentric quadriceps MVC (60°/sec)	73 ± 15	91 ± 12	< 0.001*
Concentric quadriceps MVC (180°/sec)	76 ± 14	87 ± 11	< 0.001*
Isometric quadriceps MVC	87 ± 20	93 ± 20	n.s
Isometric quadriceps RTD	82 ± 30	92 ± 25	0.033*

Data presented as mean ± standard deviation; *LSI* limb symmetry index, *MVC* maximum voluntary contractions, *RTD* rate of torque development. *p ≤ 0.05

### Effect of sex

No sex differences were found for any hamstring strength LSI measure (Table 4). Females had greater concentric quadriceps MVC and isometric RTD asymmetry, but no differences between groups were found for isometric MVC LSI (Table 4).

**Table 4:** Quadriceps and hamstring strength data for males and females collapsed across early and late rehabilitation after anterior cruciate ligament reconstruction

Strength measure	LSI (%)		Main effect
	Females	Males	Sex
Concentric hamstring MVC (60°/sec)	88 ± 12	87 ± 15	n.s
Concentric hamstring MVC (180°/sec)	90 ± 10	88 ± 14	n.s
Isometric hamstring MVC	79 ± 17	78 ± 15	n.s
Isometric hamstring RTD	85 ± 24	85 ± 49	n.s
Concentric quadriceps MVC (60°/sec)	75 ± 17	81 ± 15	0.001*
Concentric quadriceps MVC (180°/sec)	76 ± 14	83 ± 13	< 0.001*
Isometric quadriceps MVC	86 ± 20	92 ± 19	n.s
Isometric quadriceps RTD	81 ± 32	89 ± 25	0.017*

Data presented as mean ± standard deviation; *LSI* limb symmetry index, *MVC* maximum voluntary contractions, *RTD* rate of torque development. *p ≤ 0.05

### Sex by time interaction

**Fig. 1:**
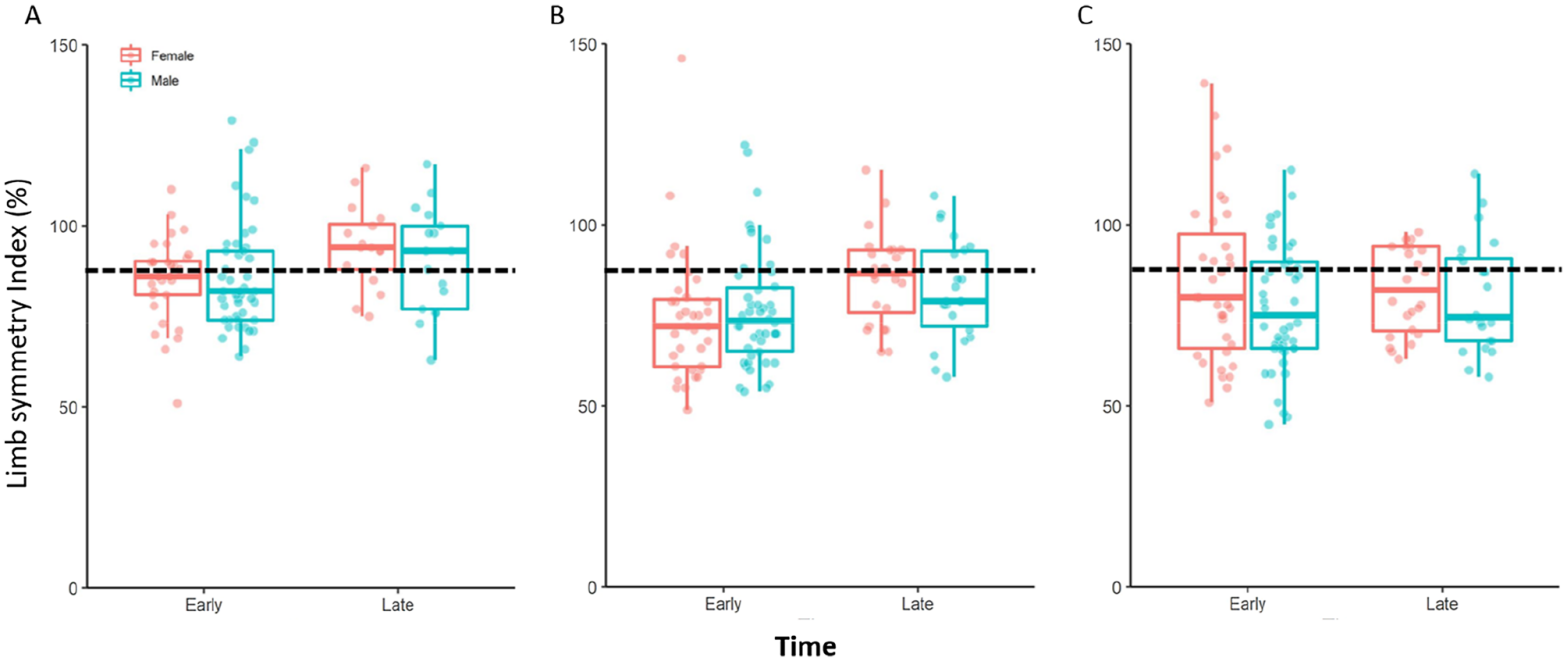
Hamstrings strength limb symmetry index (LSI) during maximal concentric strength at 60°/sec **(A)**, maximal isometric strength **(B**) and explosive isometric strength **(C)** for males and females during early and late rehabilitation after anterior cruciate ligament reconstruction. Horizontal broken line within each panel in the figure represents 90% LSI. Note: There are 2 early datapoints from male participants (LSI scores: 180 and 444) and 1 late datapoint from a female participant (LSI score: 195) that are not visible in panel C due to the scale of the Y axis

**Fig. 2:**
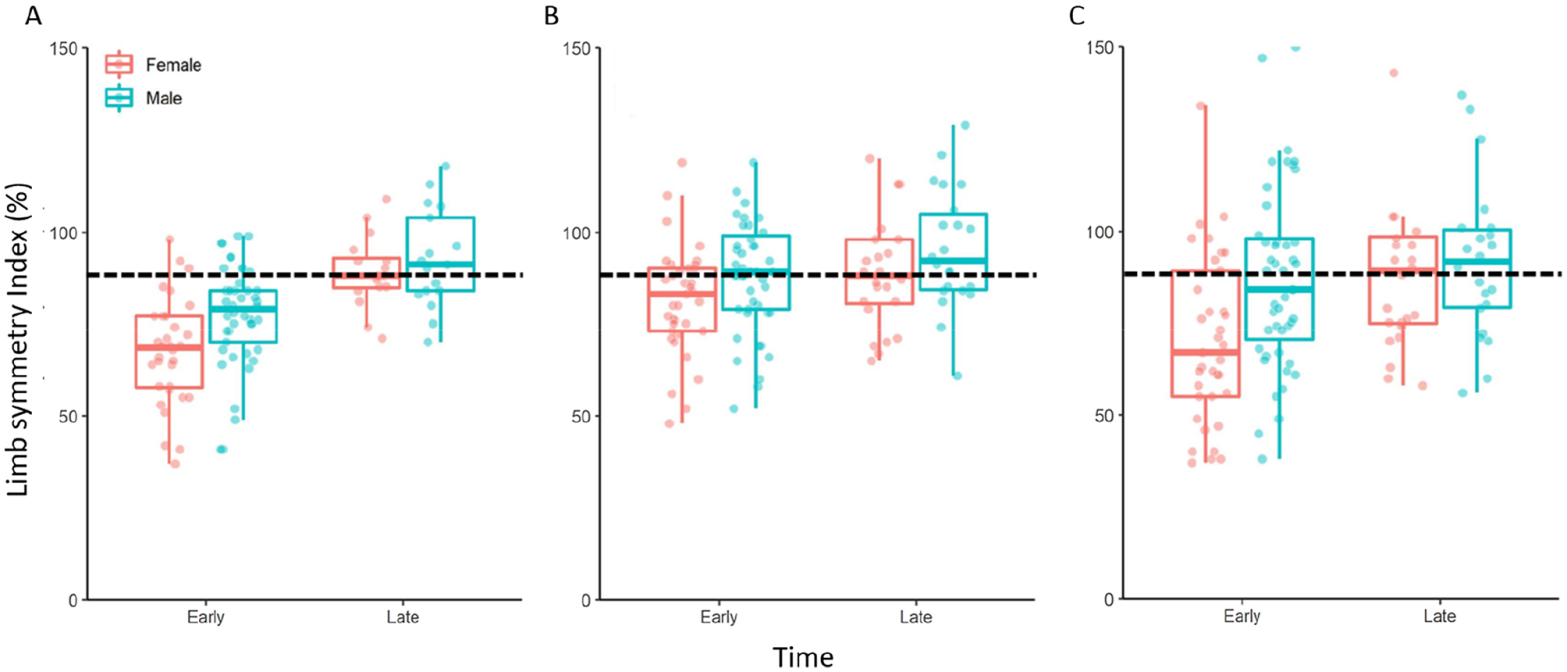
Quadriceps strength limb symmetry index (LSI) during maximal concentric strength at 60°/sec **(A)**, maximal isometric strength **(B)** and explosive isometric strength **(C)** for males and females during early and late rehabilitation after anterior cruciate ligament reconstruction. Horizontal broken line within each panel in the figure represents 90% LSI.  Note: There is 1 early male (LSI score: 160) and 1 late female (LSI score: 177) datapoint that are not visible in panel B due to the scale of the Y axis. Panel C has 2 females (LSI scores: 204 and 154) and 1 male (LSI score: 165) early datapoints and 1 female (LSI score: 177) late datapoint that are not visible due to the scale of the Y axis

No sex-by-time interactions were observed for any variables (Figs. 1 and 2, Supplementary Table 1).

## Discussion

The most important finding of this study was that maximal hamstrings strength, but not explosive hamstrings strength improved over time following ACLR using HT autografts. Additionally, analysis of the effect of sex on strength following ACLR shows that females, when compared to males, typically had larger asymmetries in measures of maximal and explosive quadriceps strength but not for hamstring strength. The results of this study shows the importance of assessing explosive hamstring strength and incorporating exercises [29] that will address these qualities following ACLR with HT grafts. Additionally, even with the use of HT grafts, maximal quadriceps strength asymmetries are more prominent in females and should be one of the main aims of rehabilitation for females after ACLR.

Based on the results of this study, patients who had ACLR with HT autografts would show maximal hamstrings strength recovery (≥ 90% LSI) during late rehabilitation period even while still having significant explosive hamstring strength asymmetry. Previous studies have also found explosive hamstring strength deficits between 3–9 months [30] and 9–12 months [31] following ACLR. However, these results were taken from only one assessment time point. This is the first study to investigate explosive hamstring strength at different time points after ACLR. It has been previously proposed that RTD is an important neuromuscular quality, especially during jumping, landing, and change-of-direction tasks [26] and is related to sports performance that require rapid movements and muscle contractions [29]. As such, explosive hamstring strength asymmetry at the time of return to sport could potentially contribute to the risk of re-injury as ACL injuries typically occurs around 50 ms after ground contact [32]. However, this is speculative and future studies should determine if there is an association between explosive strength (i.e., RTD) after ACLR and subsequent rate of re-injury.

Potential changes to hamstring function following the tendon harvest may be a contributing factor to explain the persistence of asymmetry in explosive hamstring strength found in this study [33]. Regeneration of the hamstring tendon graft likely takes somewhere between 12 and 24 months [33, 34] which could affect force-transmitting capabilities [35]. Additionally, explosive muscle strength has been correlated with muscle morphology and fiber type distribution [26] which could have been altered due to graft-site morbidity [36, 37]. The persistence of explosive hamstring strength asymmetry could also be due to the choice of exercises performed during the rehabilitation period [15]. The recovery of maximal concentric and explosive quadriceps strength with persistence of explosive hamstring strength asymmetry in this study suggest that rehabilitation might have been sufficient to address quadriceps strength but not adequate to elicit a stimulus for explosive hamstring strength recovery after ACLR with hamstring autografts [15, 38].

Analysis of the effects of sex on maximal and explosive strength following ACLR showed that males and females are typically affected similarly in the hamstrings but not in the quadriceps. This is in contrast to the results by Nielsen et al. [30] who found significant effects of sex (female more impacted than males) in maximal and explosive hamstrings strength between 3 and 9 months following ACLR. An explanation for these differences could be the larger sample size, longer duration and the frequency and timing of testing throughout ACL rehabilitation (comparing early and late rehabilitation) in this study compared to their study. On the other hand, the larger maximal and explosive quadriceps strength asymmetries found in females compared to males are in agreement with the findings of Kuenze et al. [16], who also found similar results. One thing to note, however, is that different graft types were used with their study population. In the present study, HT autografts were the graft of choice, which made the results somewhat unexpected given graft-related strength deficits are common after ACLR [9].

The exact mechanism for the quadriceps strength differences between sexes following ACLR is still inconclusive. One possible explanation could be the contribution of the inherent quadriceps morphological differences between males and females [39]. Quadriceps muscle atrophy is widely reported after ACLR and proposed to result in quadriceps weakness [40]. Given that females tend to have smaller quadriceps muscle and Type II muscle fiber CSA compared to males [39], further atrophy from the injury could potentially exacerbate these leading to greater asymmetries in quadriceps strength. This may also be considered in the hamstrings where there are differences between sexes in muscle size [41], however as reported in this study, we found no disparity in hamstring strength or RTD between males and females. Based on these findings, maximal and explosive quadriceps strength asymmetries can be expected in females following ACLR even when using HT autografts, however, the exact mechanism behind these changes are still to be determined. Overall, despite the recovery of maximal hamstrings strength throughout early and late rehabilitation, explosive hamstrings strength asymmetries tend to persist for both males and females. Sex did not influence recovery of any strength variable in this study. While females had larger quadriceps strength asymmetries overall, the rate at which this recover compared to males were similar.

The results of this study should be taken within the context of its limitations. First, the participants in this study were recruited from a single sports medicine clinic which received referrals from a small number of surgeons which ultimately reduced the heterogeneity of surgical approach. Because of this, we were unable to assess the effect that different graft types may have on the ability to restore hamstring and quadriceps strength symmetry across ACL rehabilitation and the subsequent interaction with sex. Second, there were less participants who completed late rehabilitation assessments compared to early rehabilitation assessments. This was controlled for by including participant identification number as a random factor in our statistical approach, but this approach is not infallible. To provide further information, a post hoc sensitivity analysis was conducted which included only participants (n = 42) who had early and late rehabilitation time points (Supplementary Table 2 & 3), noting that this approach has reduced statistical power compared to the main analysis. Third, a dichotomised time-based definition (early and late rehabilitation) was utilized to determine the improvement in outcomes rather than grouping based on successfully meeting pre-determined criteria to progress from the early to the late-stage rehabilitation group. However, this approach was preferred as this is more relevant for clinicians in terms of what to expect from their patients at certain periods of their rehabilitation. Furthermore, while treating time from surgery as a continuous variable (as opposed to the dichotomised early and late rehabilitation) may appear appealing, this approach is confounded by limited available data at specific time points. Lastly, participants were not matched with a healthy control group. This would have helped in identifying whether the significant asymmetries found in this study were because of the interaction of ACLR and sex over time or were simply a normal physiologic difference in strength symmetry between males and females.

## Conclusion

Following ACLR using HT autografts, explosive hamstring strength asymmetries persist despite recovery of maximal hamstring strength. These findings suggest that during rehabilitation from an ACLR, hamstring explosive strength does not recover to the same extent that maximal concentric and isometric hamstring strength does. Additionally, even with previous findings of graft-related strength deficits, females who had ACLR with HT autografts are expected to have larger quadriceps strength asymmetries compared to males.

## Supplementary Information

Below is the link to the electronic supplementary material.

Supplementary file1 (DOCX 20 KB)
