# Supplementary material for: Explosive hamstrings strength asymmetry persists despite maximal hamstring strength recovery following anterior cruciate ligament reconstruction using hamstring tendon autografts
Source: Knee Surg Sports Traumatol Arthrosc. 2022 Aug 23;31(1):299–307. doi: 10.1007/s00167-022-07096-y (PMC9859849; doi:10.1007/s00167-022-07096-y)
Supplement: Supplementary file 1 — Supplementary file1 (DOCX 20 KB) [file 167_2022_7096_MOESM1_ESM.docx]

**Supplementary Table 1** Descriptive statistics for quadriceps and hamstring strength data for males and females across early and late rehabilitation after anterior cruciate ligament reconstruction.

| Strength measure | LSI (%) | | | | |
| --- | --- | --- | --- | --- | --- |
|  | Early rehabilitation | | Late rehabilitation | | |
|  | Females (n=38) | Males  (n=51) | | Female  (n=23) | Males  (n=19) |
| Concentric hamstring MVC (60°/sec) | 85 ± 12 | 86 ± 15 | | 94 ± 11 | 90 ± 15 |
| Concentric hamstring MVC (180°/sec) | 88 ± 10 | 88 ± 14 | | 93 ± 10 | 90 ± 16 |
| Isometric hamstring MVC | 74 ± 18 | 76 ± 16 | | 85 ± 13 | 82 ± 15 |
| Isometric hamstring RTD | 84 ± 22 | 87 ± 58 | | 86 ± 26 | 80 ± 16 |
| Concentric quadriceps MVC (60°/sec) | 67 ± 15 | 77 ± 14 | | 89 ± 10 | 93 ± 14 |
| Concentric quadriceps MVC (180°/sec) | 70 ± 13 | 81 ± 13 | | 86 ± 10 | 88 ± 13 |
| Isometric quadriceps MVC | 83 ± 18 | 91 ± 21 | | 92 ± 23 | 95 ± 16 |
| Isometric quadriceps RTD | 73 ± 31 | 88 ± 27 | | 92 ± 29 | 92 ± 21 |

Data presented as mean ± standard deviation; *LSI* limb symmetry index, *MVC* maximum voluntary contractions, *RTD* rate of torque development.

**Supplementary Table 2** Post-hoc sensitivity analysis of quadriceps and hamstring strength data in early and late-stage rehabilitation after anterior cruciate ligament reconstruction collapsed across males and females who had data at both the early and late rehabilitation time points.

| Strength measure | LSI (%) | | Main effect |
| --- | --- | --- | --- |
|  | Early rehabilitation | Late rehabilitation | Time |
| Concentric hamstring MVC (60°/sec) | 85 ± 16 | 94 ± 12 | 0.003* |
| Concentric hamstring MVC (180°/sec) | 88 ± 13 | 93 ± 13 | 0.010* |
| Isometric hamstring MVC | 78 ± 20 | 85 ± 13 | 0.018* |
| Isometric hamstring RTD | 90 ± 66 | 84 ± 22 | n.s. |
| Concentric quadriceps MVC (60°/sec) | 71 ± 15 | 92 ± 12 | <0.001* |
| Concentric quadriceps MVC (180°/sec) | 76 ± 14 | 87 ± 11 | 0.001* |
| Isometric quadriceps MVC | 91 ± 22 | 95 ± 20 | n.s. |
| Isometric quadriceps RTD | 82 ± 35 | 91 ± 24 | n.s. |

Data presented as mean ± standard deviation; *LSI* limb symmetry index, *MVC* maximum voluntary contractions, *RTD* rate of torque development. **p* ≤ 0.05

**Supplementary Table 3** Post-hoc sensitivity analysis of quadriceps and hamstring strength data for males and females collapsed across early and late rehabilitation after anterior cruciate ligament reconstruction collapsed across time for participants who had data at both the early and late rehabilitation time points.

| Strength measure | LSI (%) | | Main effect |
| --- | --- | --- | --- |
|  | Females | Males | Sex |
| Concentric hamstring MVC (60°/sec) | 90 ± 13 | 89 ± 16 | n.s. |
| Concentric hamstring MVC (180°/sec) | 91 ± 10 | 90 ± 16 | n.s. |
| Isometric hamstring MVC | 83 ± 18 | 81 ± 15 | n.s. |
| Isometric hamstring RTD | 85 ± 24 | 89 ± 64 | n.s. |
| Concentric quadriceps MVC (60°/sec) | 78 ± 17 | 84 ± 17 | n.s. |
| Concentric quadriceps MVC (180°/sec) | 79 ± 13 | 84 ± 14 | 0.045* |
| Isometric quadriceps MVC | 91 ± 21 | 96 ± 21 | n.s. |
| Isometric quadriceps RTD | 84 ± 33 | 90 ± 25 | n.s. |

Data presented as mean ± standard deviation; *LSI* limb symmetry index, *MVC* maximum voluntary contractions, *RTD* rate of torque development. **p* ≤ 0.05
